# An Overview on the Anti-inflammatory Potential and Antioxidant Profile of Eugenol

**DOI:** 10.1155/2018/3957262

**Published:** 2018-10-22

**Authors:** Joice Nascimento Barboza, Carlos da Silva Maia Bezerra Filho, Renan Oliveira Silva, Jand Venes R. Medeiros, Damião Pergentino de Sousa

**Affiliations:** ^1^Department of Pharmaceutical Sciences, Universidade Federal da Paraíba, 58051-970 João Pessoa, Paraíba, Brazil; ^2^Department of Biomedicine, University Center INTA-UNINTA, 62050-130 Sobral, Ceará, Brazil; ^3^Laboratory of Pharmacology of Inflammation and Gastrointestinal Disorders-LAFIDG, Federal University of Piauí, Parnaíba, Piauí, Brazil

## Abstract

The bioactive compounds found in foods and medicinal plants are attractive molecules for the development of new drugs with action against several diseases, such as those associated with inflammatory processes, which are commonly related to oxidative stress. Many of these compounds have an appreciable inhibitory effect on oxidative stress and inflammatory response, and may contribute in a preventive way to improve the quality of life through the use of a diet rich in these compounds. Eugenol is a natural compound that has several pharmacological activities, action on the redox status, and applications in the food and pharmaceutical industry. Considering the importance of this compound, the present review discusses its anti-inflammatory and antioxidant properties, demonstrating its mechanisms of action and therapeutic potential for the treatment of inflammatory diseases.

## 1. Introduction

**Figure 1:**
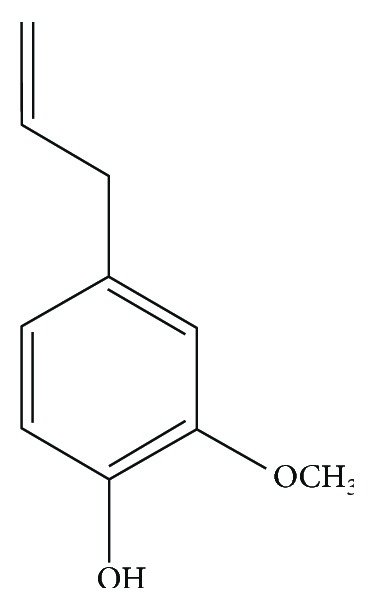
Chemical structure of eugenol.

Eugenol (4-allyl-2-methoxyphenol) is a phenolic compound from the class of phenylpropanoids and the main component of clove (*Syzygium aromaticum* (L.) Merr. & L. M. Perry.). It consists of 45–90% of its essential oil [1]. It is used in the food industry as a preservative, mainly due to its antioxidant property [2], and as a flavoring agent for foods and cosmetics [3]. It can also be found in soybean (*Glycine max* (L.) Merr.), beans [4], coffee [5], cinnamon (*Cinnamomum verum* J. Presl), basil (*Ocimum basilicum* L.) [6], “canelinha” (*Croton zehntneri* Pax et Hoffm) [7], banana [8, 9], bay laurel (*Laurus nobilis* L.), and other foods [10]. Among the plants that contain eugenol, soybeans, cloves, beans, and cinnamon also present the antioxidant activity, possibly performed by this compound and other constituents [11–14]. In addition, clove is also known by anti-inflammatory activity [15], which may be related to anti-inflammatory action of eugenol (Figure 1).

Inflammation is a complex protective response of the body against harmful agents, such as microorganisms or damaged cells [16, 17], which the biological system objective to remove harmful stimuli from the body and promote healing. However, this response needs to be controlled and last for a short period; otherwise, it may provide the appearance of pathological disorders related to the immune system [18]. Classically, inflammation can be classified in acute and chronic. The acute inflammation is an initial response, which is characterized by resident cell activation, with liberation of proinflammatory cytokines and chemokines, culminating in the recruitment of polymorphonuclear, primarily neutrophils, from the innate immune system to the injury site. This response complex act to promote cardinal signs of inflammation, such as pain, edema, and heat [19]. On the other hand, chronic inflammation is a prolonged response characterized by a gradual change in the cells type found at the inflammatory site, which over time cause both permanent damage and healing of the tissue. In both types of inflammation occur increased local blood flow, vasodilation, fluid extravasation, and liberation of proinflammatory mediators [17, 20].

The nuclear factor-kappa B (NF-*κ*B) signaling pathway is a key part of the immune response. It is essential to inflammatory processes due to its importance in the transcription of cytokines, such as tumor necrosis factor-*α* (TNF-*α*), interleukin-1*β* (IL-1*β*), interleukin-6 (IL-6), and nitric oxide (NO). Like eugenol, substances that inhibit this pathway are of interest to the pharmaceutical industry [21–23]. In general, patients with inflammatory disorders use clinically glucocorticoids or nonsteroidal anti-inflammatory drugs (NSAIDs). However, these drugs are associated with critical side effects (i.e., gastrointestinal ulcers and bleeding) and limited therapeutic efficacy, which often leads patients to abandon the treatment [24]. In this context, the pharmaceutical industry has directed efforts in the attempt to find new bioactive molecules.

Medicinal plants have been important sources of constituents with pharmacological activities. Phenylpropanoids are considered a group of secondary compounds found in a variety of plants and usually in the oxidized form, presenting a hydroxyl at the aromatic ring [25]. Studies recently demonstrated that phenylpropanoids and their synthetic derivatives have a variety of pharmacologic activities, including anti-inflammatory action [26, 27].

Several pharmacological activities have been reported to eugenol: anti-inflammatory [28], antitumor [29], antibacterial [30], antifungal [31, 32], antipyretic [33], anesthetic [34], and analgesic activities [35]. Considering the importance of eugenol as bioactive molecule and its presence in various foods and medicinal plants, this review discusses its role in the inflammatory response in experimental models, including animals and cell culture tests, demonstrating its antioxidant profile and potential therapeutical application against inflammatory diseases.

## 2. Methodology

The present review was based on the data search performed in the scientific literature database PubMed, using the publication from January 2008 to January 2018, using the following keywords: eugenol, asthma, antiasthmatic effect, allergy, antiallergic effect, inflammation, anti-inflammatory, immune response, lymphocytes, cytokines, immunoglobulins, immunoregulatory, and antioxidant.  Table 1 shows the studies reported for this review and summarizes the results obtained, indicating the dose/concentration of eugenol administered, experimental model, parameters evaluated, and biological effect.

## 3. Results and Discussion

### 3.1. Antioxidant Action of Eugenol

The free radical scavenger effect of diphenyl-1-picrylhydrazyl (DPPH) is due to the ability of certain substances to donate hydrogen, especially those with a phenolic group in their structure. Thus, eugenol's ability to sequester free radicals in the DPPH assay (IC_50_ = 11.7 *μ*g/mL), as well as to inhibit reactive oxygen species (ROS) (IC_50_ = 1.6 *μ*g/mL), H_2_O_2_ (IC_50_ = 22.6 *μ*g/mL and 27.1 *μ*g/mL), and NO (IC_50_< 50.0 *μ*g/mL) [36]. These data corroborate with other studies in which eugenol demonstrated DPPH sequestering activity with EC_50_ of 22.6 *μ*g/mL [37]. In another study, it was able to eliminate about 81% of the DPPH radicals and reduce the potency of the radicals when the concentration decreased from 1.0 *μ*M/mL to 0.1 *μ*M/mL [38]. Similar data were described in the study by Kim et al., in which eugenol performed the elimination of ABTS free radicals (76.9% at a dose of 20 *μ*g/mL) and DPPH (90.8% at a dose of 20 *μ*g/mL) in L-ascorbic acid in 76.9% and 89.9%, respectively [14].

In a comparative study of the antioxidant activity of clove and eugenol, both showed similar activities, with values of sequestering radicals DPPH and ABTS, respectively, IC_50_ = 0.3257 and 0.1595 mg/mL for the clove and of IC_50_ = 0.1967 and 0.1492 mg/mL for eugenol. Therefore, the antioxidant properties of this essential oil are related to the antioxidant action of its chemical constituent, which is eugenol [13]. The biochemical profile of this compound was confirmed in a study in which the antioxidant activity of eugenol was associated with anti-inflammatory activity. In this approach, Yogalakshmi et al. showed that pretreatment with eugenol (10.7 mg/kg.bw/day) in rats for 15 days resulted in a decrease in lipid peroxidation indices, protein oxidation, and inflammatory markers (reduction in the expression of COX-2, TNF-*α*, and IL-6) and by improving antioxidant status by maintaining antioxidants such as glutathione peroxidase (GPx), superoxide dismutase (SOD), catalase (CAT), and glutathione-S-transferase (GST) [39]. Confirming these findings, a study by Kaur et al. showed that pretreatment with eugenol in male Swiss albino mice inhibited the expression of inflammatory markers such as iNOS and COX-2 and the cytokines IL-6, TNF-*α*, and PGE2, as well as prevented the depletion of antioxidant enzymes and reduced lipid peroxidation (LPO), acting both as anti-inflammatory and antioxidant agents [40]. In fact, eugenol pretreatment, in addition to reducing inflammation caused by lung exposure to LPS, was also able to significantly improve the levels of SOD1, CAT, Gpx1, and GST. Thus, eugenol can be used as an anti-inflammatory agent, as well as protecting the damage caused by oxidative stress [41].

### 3.2. Can Eugenol Reduce the Inflammatory Response via Its Antioxidant Action?

Oxidative stress is a condition that reflect an imbalance between biological defensive and aggressive system, mediated by excessive production of reactive oxygen species (ROS), e.g., O^2−^ (superoxide radical), ^·^OH (hydroxyl radical), and H_2_O_2_ (hydrogen peroxide), in which there is an inability of the antioxidant mechanisms to neutralize them [42]. This process results in toxic effects and alterations of the normal redox state, which is associated with cellular damage and lipid peroxidation [43].

Studies have shown that inflammation and oxidative stress are interconnected phenomena, which are involved in pathological conditions as cardiovascular [44], kidney [45], liver disease [46], and cancer [47]. In this way, during inflammatory events occur exacerbated production of ROS in the damaged inflammatory tissue, which can stimulate and had a critical role in the signaling pathway for inflammatory mediators production, such as proinflammatory cytokines and chemokines, resulting in inflammatory cell migration [48].

Thus, compounds capable of modulating oxidative stress may contribute to reduce critical mediators in inflammatory events act as anti-inflammatory agents, even by indirect way. So, several research groups have demonstrated that eugenol has anti-inflammatory and antioxidant capacity, and therefore, be more effective in reducing inflammation.

### 3.3. Eugenol Reduces the Inflammatory Response and Ameliorate the Function of Specific Organ

The anti-inflammatory effect of eugenol has been investigated in the leukocytes migration using different stimuli, such as N-formyl-methionyl-leucyl-phenylalanine (fMLP), leukotriene B4 (LTB4), and carrageenan. Polymorphonuclear (PMN) recruitment to the inflammatory site occurs dependent on a complex response involving the endothelium-leukocyte interactions and subsequent extravasation to the inflamed site [49]. In this background, Estevão-Silva and colleagues [50] demonstrated that eugenol significantly decreased the *in vitro* and *in vivo* leukocytes migration in response to chemotactic factors by the modulation of rolling and adherence to perivascular tissue. In addition, the authors showed that eugenol did not induce changes in cell viability, which suggest absence of toxic effect [50].

Additionally, Pan and Dong [51], using an experimental model of allergic asthma induced by ovalbumin (OVA), demonstrated that eugenol administration inhibited the OVA-induced eosinophilia in the lung tissue, prevented the increased of IL-4 and IL-5 levels, and reduced the NF-*κ*B signalizing pathways. According to the authors, the inflammatory response reduction had a pivot role in the antiasthmatic effect of eugenol, resulting in the decrease of airway resistance (AWR) [51]. This data suggests that eugenol can be a therapeutic and strategic agent in patients with asthma.

Eugenol also has anti-inflammatory activity on lipopolysaccharide- (LPS-) induced acute lung injury. Pretreatment with eugenol inhibited the inflammatory response and leukocyte recruitment into the lung tissue by the downregulation of proinflammatory cytokines (IL-6 and TNF-*α*) expression and NF-*κ*B signaling. In addition, eugenol also increased the superoxide dismutase (SOD), catalase (CAT), glutathione peroxidase (GPx), and glutathione-S-transferase (GST), which are important antioxidative enzymes [41]. Similarly, Magalhães and colleagues [52], using an animal model of LPS-induced lung injury for 6 hours, demonstrated that eugenol significantly reduced neutrophil infiltration, TNF-*α*, and the NF-*κ*B-mediated signalizing pathway, decreasing the lung inflammation, resulting in an improved lung structure and function, which suggest an important drug to treat disorders of lung inflammatory diseases [52].

So, eugenol reduces the inflammatory response in animal model pulmonary damage caused by diesel exhaust particles. Eugenol administration reduced the pulmonary inflammation by inhibiting the PMN infiltration and apoptosis through caspase-3 cleavage but limited the effects against oxidative stress. This resulted in the improvement of airspace collapse and pulmonary mechanics, which are evaluated by pneumotachography and altered by diesel particles [53]. These data demonstrated the potential of eugenol as an agent to treat the damage effects of air pollutant exposure, by mechanisms mediated, at the last in part, of its anti-inflammatory effects.

Motteleb and colleagues (2014) conducted a study using eugenol to assess its efficacy in the prevention of liver damage in a model of ischemia and reperfusion (I/R). In this work, eugenol abolished the inflammation, reduced myeloperoxidase (MPO) activity, TNF-*α* levels, and NF-*κ*B expression, and altered oxidative marker. It also reduced malondialdehyde (MDA) and increased GSH levels. This potent effect of eugenol resulted in the amelioration of hepatic structural and functional damage [54]. Thus, eugenol reduced the liver damage by the reduction of inflammatory mediators and modulation of redox status, suggesting a possible application against hepatic I/R injury.

Eugenol also was evaluated as preventive agent against cardiac remodeling following myocardial infarction. This pathology was induced using isoproterenol, which eugenol reduced inflammatory mediator's proteins and lipid peroxidation as well as increased antioxidative enzymes markers (i.e., SOD, GPx, and GSH). In this study, eugenol reduced cardiac injury biomarkers, such as troponin-T, creatine kinase-muscle/brain (CK-MB), and LDH, resulting in the improvement of electrocardiographic and hemodynamic parameters, and great potential antithrombotic, anti-inflammatory, and anti-ischemic activities [55].

### 3.4. Eugenol Inhibits the Liberation of Inflammatory Mediators from Macrophages

Macrophage is one of the immune system cells that contribute to the production of mediators (i.e., proinflammatory cytokines and nitric oxide), which are important to cellular and vascular events during the installation and progression of inflammatory process [56]. Thus, studies have demonstrated that eugenol can modulate the macrophage functions and regulates negatively the inflammation.

Yeh and colleagues demonstrated that eugenol inhibits the inducible nitric oxide synthase (iNOS) expression from macrophages in response to LPS, culminating in the reduction of NO levels. Additionally, eugenol also reduced the TNF-*α* and IL-1*β* as well as the NF-*κ*B, ERK1/2, and p38 MAPK signaling pathways [57]. In other study, de Paula Porto and colleagues [58] also reported that eugenol promotes the downregulation of TNF-*α* in LPS-activated macrophages, which are associated with antigenotoxic activity when DNA damage was induced with doxorubicin (DXR) [58]. Thus, this data suggests that the molecular mechanisms to anti-inflammatory effects of eugenol are mediated by the regulation of inflammatory mediators production from macrophages.

### 3.5. Anti-inflammatory Effect of Eugenol Modulates the Bone Remodeling

Several research groups have described the effect of eugenol as anti-inflammatory agent and its role modulator on bone remodeling. Deepak and colleagues [59], using cell culture preexposed to RANKL (a receptor activator of NF-*κ*B ligand), demonstrated that eugenol prevented the osteoclast differentiation in a dose-dependent manner. Among the molecular mechanisms involved, the authors emphasized the downregulation of NF-*κ*B and MAPKs signaling pathways, which suggest its use in bone remodeling disorders, such as osteoporosis [59]. A recent study demonstrated that eugenol administration for twelve weeks attenuated the alveolar bone loss and remodeling associated with estrogen insufficiency using an ovariectomized (OVX) rat model, which is similar to what occurs after menopause in humans. The authors suggested that anti-inflammatory effect of eugenol had primary importance, since it was accompanied by the reduction of IL-1*β*, IL-6, and TNF-*α* levels resulting in the reduction of inflammatory cell [60].

Additionally, the effects of eugenol against inflammatory response also have been investigated in dental pulp fibroblasts from extracted third molars. During permanent teeth extractions arise postoperative complications, such as alveolar osteitis, an inflammatory condition with delayed healing and persistent pain [61]. In this context, Martínez-Herrera and colleagues [62] reported that eugenol inhibited TNF-*α* expression and NF-*κ*B signaling pathway, but not IL-1*β*, when fibroblasts was exposed to LPS, confirming its anti-inflammatory property in bone disorders. Curiously, eugenol also induced inflammatory gene mild expression in fibroblasts absence of previous inflammation [62].

### 3.6. The Antitumor Effect of Eugenol Appears to Be Mediated, in Part, by Its Anti-inflammatory Activity

**Figure 2:**
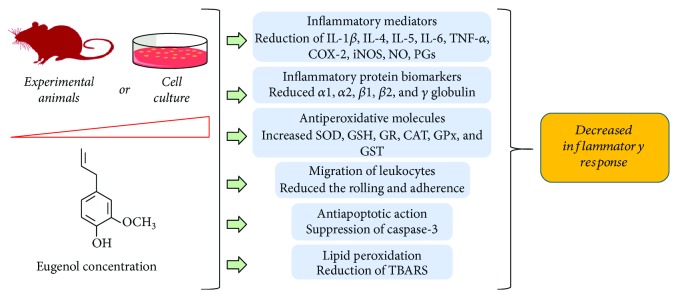
The effect of eugenol in the inflammation control.

Kaur and colleagues [40] demonstrated that eugenol prevents the 7,12-dimethylbenz[a]anthracene- (DMBA-) and 12-O-tetradecanoylphorbol-13-acetate- (TPA-) promoted skin carcinogenesis. According to the authors, the molecular mechanism of eugenol is related to its anti-inflammatory properties, since reduced proinflammatory cytokine levels (i.e., IL-6 and TNF-*α*) and inflammation enzymes marker (COX and iNOS) are associated with the modulation of redox status (Figure 2) with reduced MDA and increased antioxidative enzymes [40]. Thus, these data strongly suggest the chemotherapeutic potential of eugenol against carcinogenesis. In accordance with these data, studies have evaluated the efficacy of eugenol alone or combined with other agents. Using HeLa cells, a human cervical cancer line, Hussain and colleagues [63] demonstrated that eugenol alone promoted cell growth inhibition and increase the therapeutic efficacy when combined with gemcitabine (a standard drug). In the clinical use, it can decrease the side effects promoted by gemcitabine administration. These beneficial effects appear to be mediated by its antiapoptotic and anti-inflammatory effects, since it were associated with increased caspase-3 activity and reduction of COX-2 and IL-1*β* expression, respectively [63]. Additionally, a recent study reported that eugenol promotes cytotoxicity against breast cancer cells (TNBC) and animal model and synergistic chemotherapeutic effects with cisplatin. A key point in this effect was the inhibition of the NF-*κ*B signaling pathway, which resulted in the inhibition of the p50 and p65 subunits phosphorylation, and its consequence migration to the cellular nucleus, reducing IL-6 and IL-8 levels [23].

### 3.7. Eugenol-Based Pharmaceutical Formulation and Its Anti-inflammatory Effects

From the pharmacological potential of eugenol in the modulation of inflammation, its use has also been tested in pharmaceutical formulations. Experimentally, Esmaeili and colleagues [64], using an animal model of carrageenan-induced edema, reported that a nano-emulsion containing 1%, 2%, and 4% of eugenol reduced the edema formation and has increased efficacy when combinated with piroxicam, revealing a synergistic anti-inflammatory effect.

Additionally, a clinical dental study involving 270 patients having third molars extracted demonstrated that 0.2% chlorhexidine gel, a eugenol-based paste, reduced postoperative alveolar osteitis, pain, and time of wound healing compared to control group, but with better results when applied in two interventions [65].

### 3.8. Toxicity of Eugenol

Eugenol is known for its antioxidant, anti-inflammatory, antimicrobial, and antitumor activities; however, it may present some toxicity depending on the type of histological structure exposed to this compound and the concentration used [66]. Thus, eugenol toxicity was observed in human dental pulp fibroblasts from deciduous teeth, with DNA damage at concentrations ranging from 0.06–5.1 *μ*M, which was not observed at higher interval concentrations of 320 to 818 *μ*M [67]. Eugenol was also able to induce genotoxicity by inducing DNA damage of mouse peritoneal macrophages at all concentrations tested (0.62, 1.24, and 2.48 mg/mL). However, it has demonstrated antigenotoxic potential depending on the treatment protocol, which may be interlinked with its effect on drug metabolism [58]. Therefore, eugenol can modulate inflammatory and oxidizing processes. However, its use must be made according to the therapeutic safety evidenced in toxicity.

## 4. Conclusions

This review demonstrates that eugenol exerts a beneficial action on oxidative stress through the inhibition of enzymes and oxidative processes, which is related to the anti-inflammatory drug profile of this compound. The set of pharmacological studies reported evidences of the clinical potential of eugenol for the treatment of diseases associated with oxidative stress and inflammatory response. Considering the presence of this compound in foods and medicinal plants, the use of these vegetables can result in health benefits and consequently improvement in the quality of life. However, advanced investigations are needed to understand its metabolism in the body and the contribution of metabolites in antioxidant action and possible interactions in receptors related to inflammation.

## Figures and Tables

**Table 1:** Modulation of inflammatory response mediated by eugenol.

Experimental model	Animal and/or cells lines	Dose or concentration of eugenol	Inflammatory parameters evaluated	Biological effect	References
*In vitro* and *in vivo* leukocyte migration induced by fMLP, LTB4, and carrageenan	BALB/c mice	0.5, 1, 3, 9, or 27 *μ*g/mL 62.5, 125, or 250 mg/kg	Leukocyte migration	Decreased the number of leukocytes that rolled, adhered, and migrated to perivascular tissue	[50]
Model of allergic asthma	BALB/c mice	10 or 20 mg/kg	Cytokines (IL-4 and IL-5) levels, histological assessment, and VDUP1/NF-*κ*B signaling pathways	Inhibited OVA-induced eosinophilia, recovered IL-4 and IL-5 levels, inhibited P-I*κ*B*α*, NF-*κ*BP65, and p-NF-*κ*BP65 protein levels, and increased VDUP1 and I*κ*B*α* protein levels.	[51]
LPS-induced inflammatory reaction in acute lung injury	BALB/c mice	5 or 10 mg/kg	Activities of antioxidant enzymes (CAT, SOD, GPx, and GST) and inflammatory markers (MPO, IL-6, and TNF-*α*) and inflammatory cells recruitment	Reduced the IL-6 and TNF-*α* expression, suppressed NF-*κ*B signaling, decreased the leukocyte recruitment, and increased the protein levels (SOD, CAT, GPx, and GST)	[41]
LPS-induced lung injury	BALB/c mice	160 mg/kg body	Inflammatory cells, TNF-*α*, and NF-*κ*B levels	Reduced the neutrophil recruitment, macrophages, TNF-*α*, and NF-*κ*B expression	[52]
Diesel exhaust particles induced pulmonary damage	BALB/c mice	164 mg/kg	Amounts of polymorpho (PMN) and mononuclear cells, apoptosis, and oxidative stress	Prevented the PMN infiltration, reduced apoptosis through caspase-3 cleavage, but limited the effects on oxidative stress	[53]
Ischemia/reperfusion (I/R) injury	Wistar rats	10 or 100 mg/kg	Inflammatory markers (MPO, TNF-*α*, and NF-*κ*B p65) and oxidative stress (GSH and MDA)	Reduced MPO, TNF-*α*, NF-*κ*B, and MDA. Eugenol also increased GSH levels.	[54]
Isoproterenol-induced myocardial infarction	Wistar rats	100 mg/kg	Cells inflammatory infiltration, oxidative stress, and protein biomarker (*α*1, *α*2, *β*1, *β*2, and *γ* globulin)	Reduction of inflammatory cells infiltration and mediators proteins, increased SOD, GPx, and GSH, with reduction of TBARS	[55]
LPS-induced inflammatory signalizing	Macrophage RAW 264.7	1, 10, 50, or 100 *μ*M	Inflammatory markers (NO, TNF-*α*, IL-1*β*, and NF-*κ*B), regulatory enzymes (iNOS), and signal transduction (Akt, ERK1/2, JNK, and p38 MAPK)	Reduced NO, TNF-*α*, IL-1*β*, NF-*κ*B, and iNOS expression. Eugenol also decreased the ERK1/2 and p38 MAPK signaling pathways	[57]
LPS-activated peritoneal macrophages	BALB/c mice	0.31, 0.62, 1.24, or 2.48 *μ*g/mL	COX-2, NF-*κ*B, and TNF-*α* expression in resting macrophages	Promoted hypoexpression of TNF-*α*, but not COX-2 or NF-*κ*B	[58]
RANKL-induced osteoclast formation	RAW264.7 murine macrophages	50, 100, or 200 *μ*M	Degradation of IkB*α* and NF-*κ*B, MAPK activation	Attenuated the degradation of IkBa, activation of NF-*κ*B and MAPK pathways	[5]
Alveolar bone deformities in an ovariectomized (OVX) rodent model	Wistar rats	2.5 or 5 mg/kg	Histopathology and inflammatory mediators (IL-1*β*, IL-6, and TNF-*α*)	Reduced the inflammatory cell infiltrate, IL-1*β*, IL-6, and TNF-*α* levels	[60]
LPS-induced inflammation	Human dental pulp fibroblasts	13 *μ*M	Genes expression (NF-*κ*B, IL-1*β*, and TNF-*α*)	Inhibition of TNF-*α* expression and NF-*κ*B signaling pathway, but not IL-1*β* levels	[62]
Cutaneous chemical carcinogenesis	Swiss mice	15% (*v*/*v*)	Inflammatory markers (IL-6, TNF-*α*, PGE_2_, COX-2, and iNOS) and oxidative stress (MDA, GSH, GPx, GR, CAT, and GST)	Reduced the IL-6, TNF-*α*, PGE_2_, COX, and iNOS levels. Eugenol also decreased the MDA levels and increased the GSH content and activities of GR, CAT, GPx, and GST	[40]
Ability to interfere with cell growth	HeLa cells	300 *μ*M	Genes expression (COX-2 and IL-1*β*)	Reduced the COX-2 and IL-1*β* expression	[63]
Cisplatin-mediated toxicity	MDA-MB-231, MDA-MB-468, and BT-20 cells	0.25, 0.50, 0.75, 1.0, or 1.5 *μ*M	Gene expression (NF-*κ*B, IL-1*β*, and TNF-*α*)	Reduced NF-*κ*B, IL-1*β*, and TNF-*α* expression	[23]
Postoperative alveolar osteitis in patients having third molars extracted	Human	0.2% chlorhexidine gel, a eugenol-based paste	Postoperative pain, inflammation, infection, and wound healing	Reduced the incidence of alveolar osteitis, pain, inflammation, infection, and better wound healing compared to control group	[65]
Carrageenan-induced paw edema	Rats	1, 2, or 4%	Paw edema	Inhibited the inflammation, reducing the edema	[64]

## Abbreviations

ALDH: Aldehyde dehydrogenase
AP-I: Activating protein 1
AWR: Airway resistance
Bcl-2: B-cell lymphoma 2, apoptosis regulator
CAT: Catalase
CK-MB: Creatine kinase-muscle/brain
COX-2: Cyclooxygenase-2
DNA: Deoxyribonucleic acid
DMBA: 7,12-dimethylbenz[a]anthracene
DXR: Doxorubicin
EC_3_: Estimated concentration
fMLP: Formyl-methionyl-leucyl-phenylalanine
GPx: Glutathione peroxidase
GSH: Reduced glutathione
GST: Glutathione-S-transferase
IFN-*γ*: Interferon gamma
IL-1: Interleukin-1
IL-2: Interleukin-2
IL-4: Interleukin-4
IL-5: Interleukin-5
IL-6: Interleukin-6
IL-8: Interleukin-8
IL-18: Interleukin-18
IL-1*β*: Interleukin 1 beta
iNOS: Inducible nitric oxide synthase
LPS: Lipopolysaccharide
LTB_4_: Leukotriene B_4_
MAPK: Mitogen-activated protein kinase pathways
MDA: Malondialdehyde
MPO: Myeloperoxidase
NSAIDs: Nonsteroidal anti-inflammatory drugs
NF-*κ*B: Nuclear factor kappa B
NO: Nitric oxide
OVA: Ovalbumin
OVX: Ovariectomized
PGE: Prostaglandins
PGE2: Prostaglandins-2
PMN: Polymorphonuclear
RANKL: Receptor activator of NF-*κ*B ligand
SI: Stimulation indices
SOD: Dismutase
TH1: T helper type 1
TH2: T helper type 2
TNF-*α*: Tumor necrosis factor *α*
TNBC: Cytotoxicity against breast cancer cells
TPA: 12-O-tetradecanoylphorbol-13-acetate
TRAP: Acid phosphatase assay.
